# Medico-legal liability of injuries arising from laryngoscopy

**DOI:** 10.1017/S0022215123001986

**Published:** 2024-05

**Authors:** Christian G Fritz, Stylianos D Monos, Dominic Romeo, Anne Lowery, Katherine Xu, Joshua Atkins, Karthik Rajasekaran

**Affiliations:** 1 Department of Otorhinolaryngology – Head & Neck Surgery, University of Pennsylvania, Philadelphia, USA; 2 Lewis Katz School of Medicine at Temple University, Philadelphia, Pennsylvania, USA; 3 Perelman School of Medicine at the University of Pennsylvania, Philadelphia, USA; 4 Department of Anesthesiology and Critical Care, University of Pennsylvania, Philadelphia, USA; 5 Leonard Davis Institute of Health Economics, University of Pennsylvania, Philadelphia, PA, USA

**Keywords:** Larynx, tooth injuries, anesthesia, legal case, legal liability, jurisprudence

## Abstract

**Objective:**

Dental and mucosal injuries from laryngoscopy in the peri-operative period are common medico-legal complaints. This study investigated lawsuits arising from laryngoscopy.

**Methods:**

Westlaw, a legal database containing trial records from across the USA, was retrospectively reviewed. Plaintiff and/or defendant characteristics, claimed injuries, legal outcomes and awards were extracted.

**Results:**

Of all laryngoscopy-related dental or mucosal injuries brought before a state or federal court, none (0 per cent) resulted in a defence verdict against the provider or monetary gain for the patient. Rulings in the patient's favour were observed only when laryngoscopy was found to be the proximate cause of multiple compounding complications that culminated in severe medical outcomes such as exsanguination, septic shock or cardiopulmonary arrest.

**Conclusion:**

Proper laryngoscopy technique and a robust informed-consent process that accurately sets patients' expectations reduces litigation risk. Future litigation pursuits should consider the low likelihood of malpractice allegation success at trial.

## Introduction

Laryngoscopy is a procedure that enables visualisation of the larynx. It is instrumental in the setting of endotracheal intubation and surgical procedures of the airway. As with any procedure, complications may arise. Mucosal injury involving the lips or angles of the mouth have been reported in up to 75 per cent of suspension laryngoscopy cases.^1^ Moreover, dental injury has a highly variable and provider-dependent incidence, which may range from 25–39 per cent to as low as 0.02–0.1 per cent.^2–15^ It is well established that dental injury occurring in the peri-operative period is the most common medico-legal complaint against anaesthesiologists, comprising more than one-third of all legal claims within the specialty.^9,12,16^

In addition to allegations involving dentition, there are also less-common complications directly mediated by the laryngoscope that may include gingival trauma in edentulous patients;^17^ injury to the pharyngeal arches and tonsillolingual sulci;^18^ lingual, glossopharyngeal and hypoglossal nerve injury;^1,19^ and ischaemic injury of the tongue.^20,21^ Although the majority of injuries caused by the laryngoscopy are limited in severity, any payment made to patients may be reported and become part of the National Practitioner Data Bank.^22^ This malpractice reporting is known to affect future job prospects and reduce clinical productivity.^23–26^ Therefore, a clear understanding of these allegations and legal outcomes is of the utmost importance.

Prior reports utilising the Westlaw legal database have addressed related topics that are more rare and severe, such as iatrogenic dysphonia,^27^ laryngotracheal stenosis,^28^ and vocal fold paralysis.^29^ Indeed, such reviews have established the Westlaw database as a representative source of information within the field of otolaryngology.

Capturing information for approximately 20 patients is not uncommon for articles addressing rare events with few corresponding malpractice cases.^30–32^ Given that dental and mucosal injuries are much more prevalent, understanding the legal consequences of these complications may be relevant to a larger population of interested parties, both plaintiffs and defendants. To the best of our knowledge, this is the first report in the literature to use a legal database to retrospectively assess the outcomes of medical malpractice cases involving laryngoscopy in the USA.

The current study seeks to understand the clinical circumstances, medical sequelae, and financial implications of laryngoscopy-associated injury litigation. This pursuit may inform recommendations that enhance patient safety and provide new insights for providers facing a lawsuit alleging negligence during laryngoscopy. Findings from this analysis could also serve as an educational tool for anaesthesiology and otolaryngology residents, nurse anaesthetists in training, and others learning how safely to manipulate the airway.

## Materials and methods

An online legal research database (Westlaw, West Publishing Co, St Paul, MN), was used to extract details from pertinent cases for the purposes of this retrospective analysis. This subscription-based database is widely used by legal professionals in the USA to understand trial precedent arising from federal and state court cases. Jury verdict and settlement reports were reviewed for relevance based on the following combination of terms: ‘malpractice’ AND (‘laryngoscopy’ OR ‘panendoscopy’ OR ‘microlaryngoscopy’) OR ‘laryngoscope’ OR ‘McGrath’ OR ‘C-MAC’ OR ‘intubation’ OR ‘intubate’ AND ‘tongue’ OR ‘lip’ OR ‘mouth’ OR ‘throat’ OR ‘gum’ OR ‘mucosa’ OR ‘tooth’ OR ‘teeth’ OR ‘dental’ OR ‘bridge.’ This study was approved by the Institutional Review Board (IRB) of University of Pennsylvania Health System.

From the 155 initial results, cases were excluded for the following reasons: incidental mention of keywords (i.e. laryngoscopy was not an alleged mechanism of injury in litigation) (104) and duplicate cases (31). The remaining 20 verdict and settlement reports were comprehensively evaluated for several details including: year, state, patient demographics, defendant specialty, court type, procedure performed, claimed injury, case outcome, damage amount awarded, and allegations involving either incomplete informed consent, requirement of reparative procedures, functional deficits incurred, psychiatric and/or psychological sequelae, depression and/or loss of enjoyment of life, and other alleged causes of negligence. Data collection was completed in October 2022. Descriptive statistical analyses were performed using SPSS software (version 24, IBM).

## Results and analysis

Twenty lawsuits that took place between 1990 and 2018 met inclusion criteria. Cases were stratified into dental and/or mucosal injuries (*n* = 12, 60 per cent) and other laryngoscopy complications involving the tongue, prosthodontic device, pharynx or multifactorial (*n* = 8, 40 per cent) (Table 1). The majority of plaintiffs were female and middle-aged. Implicated providers were most commonly anaesthesiologists and Certified Registered Nurse Anaesthetists (CRNAs). The most frequently cited allegations involved medical negligence (75.0 per cent), improper history and physical exam (33.3 per cent), and incomplete informed consent (16.7 per cent).

**Table 1.** Comparison of case characteristics, mucosal and/or dental injuries *vs* other laryngoscopy complications

	Mucosal and/or dental injuries (*n* = 12)	Other laryngoscopy complications (*n* = 8)
Plaintiff gender (*n* (%))		
– Male	2 (16.7)	1 (12.5)
– Female	10 (83.3)	7 (87.5)
Plaintiff age years, mean (SD)	54.6 (12.9)	60.0 (17.8)
Trial verdict (*n* (%))		
– Defence	11 (91.7)	2 (25.0)
– Plaintiff	0 (0.0)	6 (75.0)
– Settlement	1 (83.3)*	0 (0.0)
Award amount		
– Range	$0–0	$0–1.22 million
– Mean	$0	$592,119
Defendant specialty (*n* (%))	
– Anaesthesiology	8 (66.7)	4 (50.0)
– CRNA	3 (25.0)	2 (25.0)
– Emergency medicine	1 (8.3)	1 (12.5)
– Internal medicine	0 (0.0)	1 (12.5)
– Otolaryngology	0 (0.0)	0 (0.0)
Allegations (*n* (%))		
– Medical negligence	9 (75.0)	7 (87.5)
– Improper history & physical exam	4 (33.3)	1 (12.5)
– Incomplete informed consent	2 (16.7)	2 (25.0)
– Required reparative procedures	1 (8.3)	0 (0.0)
– Psychiatric and/or psychological sequelae	1 (8.3)	1 (12.5)
– Pain and suffering	1 (8.3)	3 (37.5)
– Unnecessary procedures	1 (8.3)	0 (0.0)
– Lost wages	1 (8.3)	1 (12.5)
– Excessive force to restrain	1 (8.3)	0 (0.0)
– Unqualified provider	0 (0.0)	1 (12.5)
– Undue medical expenses	0 (0.0)	2 (25.0)
– Disfigurement	0 (0.0)	1 (12.5)

*Arbitration with a known award of $0 and unknown settlement amount; SD = standard deviation; CRNA = Certified Registered Nurse Anaesthetist.

Specific claimed injuries are detailed in Table 2. All cases were related to laryngoscope use during pre-operative intubation procedures. No case of dental and/or mucosal injury led to a verdict in favour of the plaintiff. Among all captured dental injury cases, the most severe involved avulsion of three teeth and fracture of a fourth tooth. The least severe injury brought before the courts was a laryngoscope-mediated mucosal laceration that allegedly caused a throat infection. Taken together, the average pay-out for dental and/or mucosal injury was $0.

**Table 2.** Laryngoscopy litigation outcomes and case characteristics

Outcome	State	Plaintiff award ($)	Site of injury	Claimed injuries	Case reference
Defence verdict	VA	0	Teeth	Damage to molars that required dental care and long-term tongue numbness	Sturgis *vs* Bajit
Defence verdict	MO	0	Teeth	Damage to dental bridge	Delunas *vs* Creve Coeur Surgery Center
Defence verdict	VA	0	Teeth	Multiple tooth fractures and ‘mouth nerve damage’	Baj *vs* Sturg
Defence verdict	AL	0	Teeth	Teeth broken and displaced	Shelly Purifoy Nelson *vs* Cullman Regional Medical Center, Inc and Robin T Hall, MD
Defence verdict	NY	0	Teeth	Dislodgment of three teeth and damage to a fourth tooth	Silverman *vs* Nyack Hospital *et al*.
Defence verdict	NJ	0	Teeth	Loss of a tooth, post-traumatic stress disorder, torn rotator cuff, and contusions to tongue	Petrie *vs* Naik, MD; Voros, MD; University of Medicine and Dentistry of New Jersey
Defence verdict		0	Teeth	Loss of two teeth and excessive use of force in opening mouth	Labella *vs* New York Hospital Medical Center of Queens; Treiber, MD
Settlement	NY	0	Teeth	Loss of teeth	Roper *vs* Tumaian; Taquinta; Delaware County Memorial Hospital
Defence verdict	PA	0	Mucosa	Laceration of the right soft palette and tonsil, requiring an emergency tonsillectomy and cancellation of planned surgery	Holley *vs* Dekalb Anesthesia Associates PA; Dunac, MD
Defence verdict	GA	0	Mucosa	Wound from laryngoscope assisted intubation caused severe throat infection	Brukner *vs* Zerwas
Defence verdict	MI	0	Mucosa	Laceration of soft palate which required an emergency tracheostomy and surgical repair	Benish *vs* Prokott, DO; Dayman, CRNA; Great Lakes Anesthesia Associates
Defence verdict	MI	0	Mucosa	Damage to pharynx, respiratory distress, dysphagia, throat pain, hoarseness, and a jagged laceration of the right soft palate	Tan, MD *vs* Soto, MD
Defence verdict	FL	0	Tongue	Severe tongue trauma resulting in gangrenous change and amputation of anterior two-thirds of tongue	Edith Clute *vs* Dagan Payne Dalton, MD, Heart of Florida Hospital Association, Inc
Defence verdict	NY	0	Prosthodontic device	Laryngoscope dislodged 5-tooth dental bridge	Schneider *vs* Maimonides Medical Center; Janardhan, MD; Shah, MD
Plaintiff verdict	NJ	7,895	Prosthodontic device	Laryngoscope dislodged dental bridge, which resulted in fracture of two natural teeth and malocclusion	Quinn *vs* Degroot *et al*.
Plaintiff verdict	FL	400,000	Multifactorial	Tooth loss after excessive prescription of sedatives causing respiratory failure and exacerbation of underlying conditions	Ann Giri Shevetz *vs* Mark Schor, MD
Plaintiff verdict	IN	938,800	Pharynx	Laryngoscope displaced dentures, pharynx injury, internal bleeding, aspiration, lung collapse, shock, and death	Creviston *vs* St Mary Medical Center
Plaintiff verdict	IL	1,061,842	Pharynx	Catastrophic exsanguination due to a laryngoscope blade impaling a known congenital vascular malformation resulting in death	Tadros *vs* Hospital of Cook County; Mcpencow, MD; Konefal, MD
Plaintiff verdict	IL	1,103,413	Pharynx	Perforation of the pharynx, sore throat, difficulty swallowing, and subcutaneous emphysema	Cooper *vs* Paisansathan
Plaintiff verdict	KS	1,225,000	Tongue	Tongue laceration, swelling of the tongue, airway obstruction, cardiopulmonary arrest, anoxic brain injury, aspiration pneumonia, septic shock, and death.	Newsome *vs* Anesthesia Associates of Kansas City

MD = Doctor of medicine; DO = Doctor of osteopathic medicine; CRNA - Certified registered nurse anesthetist

Cases culminating in a plaintiff verdict or award were heterogeneous in their site of injury, and were not limited to dental or mucosal structures. These cases tended to have multiple compounding complications that resulted in severe morbidity. The average award for plaintiff verdicts was $592,119 (range $0–1.22 million). Of those cases returning a plaintiff verdict, three of six (50.0 per cent) resulted in patient death. For the remaining three non-morbid cases, there was one multifactorial incident in which an internal medicine physician was held liable for excessive prescription of sedatives that caused respiratory failure necessitating intubation, which was complicated by dental avulsion from laryngoscope use. The remaining two plaintiff–verdict cases involved a transmural pharynx perforation and prosthetic bridge disruption, the latter of which resulted in the smallest payment awarded ($7,895).

## Discussion

Malpractice litigation is responsible for increased health care costs and is often viewed as adversarial by physicians.^33–36^ Regardless of outcome, litigation can affect a practitioner's reputation among peers and patients.^37^ Given that dental and mucosal injuries are almost universally presented as possible complications of laryngoscopy during the consent process, some providers may find it perplexing that these complaints comprise more than one-third of all lawsuits in the field of anaesthesiology.^9,12,16^ In many of these cases, poor documentation of existing problems with mucosa or dentition prior to laryngoscopy make such cases viable. In this report, allegations of medical malpractice that culminated in a lawsuit brought before a court of law were comprehensively analysed to reveal new insights into a key gap in the literature.

We report that dental and/or mucosal injury cases were unanimously ruled in favour of the defendant medical provider with none resulting in payment awards to a plaintiff. This outcome was consistently observed among both anaesthesiologist and certified registered nurse anaesthetist defendants. While laryngoscopy is commonly performed by otolaryngologists, there was no documented lawsuit brought before the court involving laryngoscopy-associated injury alleged against an otolaryngologist. This could be due to a lower volume of laryngoscopies performed among otolaryngologists. Although anaesthesia providers do perform the majority of these procedures in the peri-operative period, the fact that no allegation involved an otolaryngologist could suggest variability in technique and strain forces utilised between different specialties. Simulation-based exercises are one effective method to help minimise laryngoscope-associated injuries. Such educational resources may prove useful in training clinicians on best practices for laryngoscopy.^38–40^ Regardless of the specialty involved, a dental- or oral-surgery consultation should be sought upon recognition of any iatrogenic dental injury.

In addition to dental and mucosal injuries, it is also important to be mindful of delicate soft-tissue structures in contact with the laryngoscope. Indeed, the highest award ($1,225,000) was returned for a case in which a GlideScope® caused a tongue laceration, which lead to swelling of the tongue, airway obstruction, cardiopulmonary arrest, anoxic brain injury, aspiration pneumonia, septic shock and death.

Fortunately, severe complications directly attributable to laryngoscopy were rare. When such events did occur, they were commonly due to unforeseen anatomical circumstances. For instance, one case involved displacement of dentures causing transmural pharynx perforation, internal bleeding, aspiration, lung collapse, shock and death. Another case involved exsanguination due to disruption of a known congenital vascular malformation. As a result of the aforementioned cases, improper history and physical examination was the second most commonly cited allegation among all cases. This highlights the need for continued focus on peri-operative evaluation that adheres to previously established American Society of Anesthesiologists guidelines and consensus statements (https://www.asahq.org/standards-and-guidelines).

Although the field of anaesthesiology was implicated in the majority of laryngoscope-associated malpractice allegations, the authors think that the results of this report are of greatest relevance to otolaryngologists. In contrast to laryngoscopy performed by anaesthesia providers, laryngoscopy performed by otolaryngologists is often prolonged in duration and holds more potential for complications. Otolaryngologists routinely perform in-office flexible laryngoscopy, indirect mirror laryngoscopy, laryngeal stroboscopy, direct laryngoscopy and suspension laryngoscopy. When considering the high combined prevalence of these procedures, the fact that no laryngoscope injury malpractice case tried to date has implicated an otolaryngologist suggests that laryngoscopy procedures are highly safe and low-risk when performed by an otolaryngologist–head and neck surgeon. This represents an important counselling point that may provide a source of reassurance to patients who are averse to laryngoscopy. While the procedure may be uncomfortable and fear-inducing in some cases, patients may find it reassuring to know that no patient has ever taken their otolaryngologist to court over complications arising from a procedure to visualise the larynx.

Although claims of dental injury are very common, formal lawsuits brought before the courts are exceedingly rare. While prior reports have documented thousands of such dental injury claims,^41,42^ these tend to culminate in out-of-court settlements, arbitration or a summary judgement motion without a trial. The present study is unique in that it is the first to analyse lawsuit outcomes as determined by objective review via trial by jury in a federal or state courtroom. A recent comprehensive review of a French legal database identified just 24 lawsuits involving peri-operative dental injuries.^43^ This relatively low case number highlights the rarity of these trials and reaffirms our assertation that the present review successfully captures all available court records on laryngoscope-mediated dental injuries. This report, therefore, may represent a valuable resource for predicting future litigation outcomes and may be referenced by expert witnesses under oath as precedent to justify verdicts.

- Dental and mucosal injuries caused by laryngoscopy procedures in the peri-operative period are common sources of frustration for patients
- No malpractice court trial pertaining to laryngoscopy-related dental or mucosal injuries has successfully proven this allegation in court
- No allegations were levied against otolaryngologists; primary defendants were most commonly anaesthesiologists (60 per cent) and certified registered nurse anaesthetists (20 per cent)
- Given that laryngoscopy complications comprise more than one-third of all lawsuits against anaesthesiologists, the findings of this report are of interest to a large group of providers

There are several limitations to this study. Included cases were limited to those attributable to laryngoscopy, but not the process of intubation, which can be an independent source of litigation with a distinct set of injuries. Moreover, the Westlaw database only contains jury verdict reports from federal or state courts, thereby failing to capture cases that do not progress to this stage. Verdict and settlement summaries are also highly heterogeneous sources of information with variable degrees of information disclosed, as deemed necessary by attorneys privy to the case. Finally, most malpractice litigation does not go to trial, with up to 85 per cent of cases being dismissed in a summary judgment or resolved with an out-of-court settlement.^44,45^ Because the cost of defending cases in court can be vastly disproportionate to the cost of dental repairs, many dental claims may be settled informally.^11^ Taken together, the cases presented herein likely represent a small subset of all allegations surrounding laryngoscopy-associated injuries. Despite these limitations, our analysis provides important insights that can be used to better understand laryngoscopy litigation, inform educational endeavours and improve patient care.

## Conclusion

Among laryngoscopy-associated injury cases, complications involving dental and mucosal structures in the peri-operative period were the most commonly litigated cases. No case limited to dental or mucosal injury resulted in monetary gain for the patient. Given that laryngoscopy complications may comprise more than one-third of all legal claims against anaesthesiologists, these findings are of interest to a relatively large group of providers and offer evidence-based reassurance that lawsuits will most likely return a defence verdict if the case is tried before a jury. Efforts should be made to perform laryngoscopy with proper caution and thoroughly discuss risks delineated on consent forms to ensure that patients are fully aware of potential complications.

## References

[1] Klussmann JP, Knoedgen R, Wittekindt C, Damm M, Eckel HE. Complications of suspension laryngoscopy. Ann Otol Rhinol Laryngol 2002;111:972–6 12450169 10.1177/000348940211101104

[2] Holzer JF. Analysis of anesthetic mishaps. Current concepts in risk management. Int Anesthesiol Clin 1984;22:91–116 6724714 10.1097/00004311-198408000-00009

[3] Burton JF, Baker AB. Dental damage during anaesthesia and surgery. Anaesth Intensive Care 1987;15:262–8 2889396 10.1177/0310057X8701500304

[4] Lockhart PB, Feldbau EV, Gabel RA, Connolly SF, Silversin JB. Dental complications during and after tracheal intubation. J Am Dent Assoc 1986;112:480–3 2871061 10.14219/jada.archive.1986.0035

[5] Chopra V, Bovill JG, Spierdijk J. Accidents, near accidents and complications during anaesthesia. A retrospective analysis of a 10-year period in a teaching hospital. Anaesthesia 1990;45:3–6 2316834 10.1111/j.1365-2044.1990.tb14492.x

[6] Bucx MJ, Snijders CJ, van Geel RT, Robers C, van de Giessen H, Erdmann W et al. Forces acting on the maxillary incisor teeth during laryngoscopy using the Macintosh laryngoscope. Anaesthesia 1994;49:1064–70 7864323 10.1111/j.1365-2044.1994.tb04358.x

[7] Brosnan C, Radford P. The effect of a toothguard on the difficulty of intubation. Anaesthesia 1997;52:1011–14 9370848 10.1111/j.1365-2044.1997.221-az0355.x

[8] Warner ME, Benenfeld SM, Warner MA, Schroeder DR, Maxson PM. Perianesthetic dental injuries: frequency, outcomes, and risk factors. Anesthesiology 1999;90:1302–5 10319777 10.1097/00000542-199905000-00013

[9] Owen H, Waddell-Smith I. Dental trauma associated with anaesthesia. Anaesth Intensive Care 2000;28:133–45 10788963 10.1177/0310057X0002800202

[10] Cass NM. Medicolegal claims against anaesthetists: a 20 year study. Anaesth Intensive Care 2004;32:47–58 15058121 10.1177/0310057X0403200108

[11] Givol N, Gershtansky Y, Halamish-Shani T, Taicher S, Perel A, Segal E. Perianesthetic dental injuries: analysis of incident reports. J Clin Anesth 2004;16:173–6 15217655 10.1016/j.jclinane.2003.06.004

[12] Yasny JS. Perioperative dental considerations for the anesthesiologist. Anesth Analg 2009;108:1564–73 19372337 10.1213/ane.0b013e31819d1db5

[13] Mourão J, Neto J, Viana JS, Carvalho J, Azevedo L, Tavares J. A prospective non-randomised study to compare oral trauma from laryngoscope versus laryngeal mask insertion. Dent Traumatol 2011;27:127–30 21281439 10.1111/j.1600-9657.2010.00947.x

[14] Mourão J, Neto J, Luís C, Moreno C, Barbosa J, Carvalho J et al. Dental injury after conventional direct laryngoscopy: a prospective observational study. Anaesthesia 2013;68:1059–65 24047290 10.1111/anae.12342

[15] Kim HJ, Lee JM, Bahk JH. Assisted head extension minimizes the frequency of dental contact with laryngoscopic blade during tracheal intubation. Am J Emerg Med 2013;31:1629–33 24041638 10.1016/j.ajem.2013.08.019

[16] Cook TM, Scott S, Mihai R. Litigation related to airway and respiratory complications of anaesthesia: an analysis of claims against the NHS in England 1995–2007. Anaesthesia 2010;65:556–63 20345420 10.1111/j.1365-2044.2010.06331.x

[17] Mourão J, Moreira J, Barbosa J, Carvalho J, Tavares J. Soft tissue injuries after direct laryngoscopy. J Clin Anesth 2015;27:668–71 26391674 10.1016/j.jclinane.2015.07.009

[18] Pham Q, Lentner M, Hu A. Soft palate injuries during orotracheal intubation with the videolaryngoscope. Ann Otol Rhinol Laryngol 2017;126:132–7 27831515 10.1177/0003489416678008

[19] Rosen CA, Andrade Filho PA, Scheffel L, Buckmire R. Oropharyngeal complications of suspension laryngoscopy: a prospective study. Laryngoscope 2005;115:1681–4 16148717 10.1097/01.MLG.0000175538.89627.0D

[20] Lindemann TL, Kamrava B, Sarcu D, Soliman AMS. Tongue symptoms, suspension force and duration during operative laryngoscopy. Am J Otolaryngol. 2020;41:102402 31982210 10.1016/j.amjoto.2020.102402

[21] Onal M, Colpan B, Elsurer C, Bozkurt MK, Onal O, Turan A. Is it possible that direct rigid laryngoscope-related ischemia-reperfusion injury occurs in the tongue during suspension laryngoscopy as detected by ultrasonography: a prospective controlled study. Acta Otolaryngol 2020;140:583–8 32223688 10.1080/00016489.2020.1743353

[22] Kain ZN. The National Practitioner Data Bank and anesthesia malpractice payments. Anesth Analg 2006;103:646–9 16931675 10.1213/01.ane.0000226218.55224.53

[23] Shanafelt TD, Balch CM, Bechamps G, Russell T, Dyrbye L, Satele D et al. Burnout and medical errors among American surgeons. Ann Surg 2010;251:995–1000 19934755 10.1097/SLA.0b013e3181bfdab3

[24] Balch CM, Oreskovich MR, Dyrbye LN, Colaiano JM, Satele DV, Sloan JA et al. Personal consequences of malpractice lawsuits on American surgeons. J Am Coll Surg 2011;213:657–67 21890381 10.1016/j.jamcollsurg.2011.08.005

[25] Chen KY, Yang CM, Lien CH, Chiou HY, Lin MR, Chang HR et al. Burnout, job satisfaction, and medical malpractice among physicians. Int J Med Sci 2013;10:1471–8 24046520 10.7150/ijms.6743 PMC3775103

[26] Seabury SA, Chandra A, Lakdawalla DN, Jena AB. On average, physicians spend nearly 11 percent of their 40-year careers with an open, unresolved malpractice claim. Health Aff *(*Millwood*)* 2013;32:111–19 23297278 10.1377/hlthaff.2012.0967 PMC6385890

[27] Ta JH, Liu YF, Krishna P. Medicolegal aspects of iatrogenic dysphonia and recurrent laryngeal nerve injury. Otolaryngol Head Neck Surg 2016;154:80–6 26419840 10.1177/0194599815607220

[28] Svider PF, Pashkova AA, Husain Q, Mauro AC, Eloy JD, Baredes S et al. Determination of legal responsibility in iatrogenic tracheal and laryngeal stenosis. Laryngoscope 2013;123:1754–8 23404544 10.1002/lary.23997

[29] Swonke ML, Shakibai N, Chaaban MR. Medical malpractice trends in thyroidectomies among general surgeons and otolaryngologists. OTO Open. 2020;4:2473974X20921141 10.1177/2473974X20921141 PMC7223205 32435722

[30] Kovalerchik O, Mady LJ, Svider PF, Mauro AC, Baredes S, Liu JK et al. Physician accountability in iatrogenic cerebrospinal fluid leak litigation. Int Forum Allergy Rhinol 2013;3:722–5 23536469 10.1002/alr.21169

[31] Svider PF, Blake DM, Sahni KP, Folbe AJ, Liu JK, Baredes S et al. Meningitis and legal liability: an otolaryngology perspective. Am J Otolaryngol 2014;35:198–203 24074731 10.1016/j.amjoto.2013.07.001

[32] Winford TW, Wallin JL, Clinger JD, Graham AM. Malpractice in treatment of sinonasal disease by otolaryngologists: a review of the past 10 years. Otolaryngol Head Neck Surg 2015;152:536–40 25573677 10.1177/0194599814566787

[33] Annas GJ. Doctors, patients, and lawyers—two centuries of health law. N Engl J Med 2012;367:445–50 22853015 10.1056/NEJMra1108646

[34] Brooks RG, Menachemi N, Hughes C, Clawson A. Impact of the medical professional liability insurance crisis on access to care in Florida. Arch Intern Med 2004;164:2217–22 15534157 10.1001/archinte.164.20.2217

[35] Mello MM, Kachalia A, Goodell S. Medical malpractice – April 2011 update. Synth Proj Res Synth Rep 2011;21(suppl 1):72097 22052245

[36] Nahed BV, Babu MA, Smith TR, Heary RF. Malpractice liability and defensive medicine: a national survey of neurosurgeons. PLoS One 2012;7:e39237 22761745 10.1371/journal.pone.0039237 PMC3382203

[37] Burkle CM, Martin DP, Keegan MT. Which is feared more: harm to the ego or financial peril? A survey of anesthesiologists’ attitudes about medical malpractice. Minn Med 2012;95:46–50 23094415

[38] Batchelder AJ, Steel A, Mackenzie R, Hormis AP, Daniels TS, Holding N. Simulation as a tool to improve the safety of pre-hospital anaesthesia—a pilot study. Anaesthesia 2009;64:978–83 19686483 10.1111/j.1365-2044.2009.05990.x

[39] Hodd JAR, Doyle DJ, Gupta S, Dalton JE, Cata JP, Brewer EJ et al. A mannequin study of intubation with the AP advance and GlideScope Ranger videolaryngoscopes and the Macintosh laryngoscope. Anesth Analg 2011;113:791–800 21890882 10.1213/ANE.0b013e3182288bda

[40] Vanderbilt AA, Mayglothling J, Pastis NJ, Franzen D. A review of the literature: direct and video laryngoscopy with simulation as educational intervention. Adv Med Educ Pract 2014;5:15–23 24501548 10.2147/AMEP.S51963 PMC3912064

[41] Christensen RE, Baekgaard JS, Rasmussen LS. Dental injuries in relation to general anaesthesia—a retrospective study. Acta Anaesthesiol Scand 2019;63:993–1000 31016717 10.1111/aas.13378

[42] Kotani T, Inoue S, Kawaguchi M. Perioperative dental injury associated with intubated general anesthesia. Anesth Prog 2022;69:3–9 10.2344/anpr-68-03-02 PMC8985457 35377930

[43] Diakonoff H, De Rocquigny G, Tourtier JP, Guigon A. Medicolegal issues of peri-anaesthetic dental injuries: a 21-years review of liability lawsuits in France. Dent Traumatol 2022;38:391–6 35639817 10.1111/edt.12770 PMC9539868

[44] Jena AB, Seabury S, Lakdawalla D, Chandra A. Malpractice risk according to physician specialty. N Engl J Med 2011;365:629–36 21848463 10.1056/NEJMsa1012370 PMC3204310

[45] Jena AB, Chandra A, Lakdawalla D, Seabury S. Outcomes of medical malpractice litigation against US physicians. Arch Intern Med 2012;172:892–4 22825616 10.1001/archinternmed.2012.1416

